# US states that mandated COVID-19 vaccination see higher, not lower, take-up of COVID-19 boosters and flu vaccines

**DOI:** 10.1073/pnas.2403758121

**Published:** 2024-09-30

**Authors:** Jack Fitzgerald

**Affiliations:** ^a^Department of Ethics, Governance, and Society, Vrije Universiteit Amsterdam, School of Business and Economics, Amsterdam 1081HV, The Netherlands

Rains and Richards (1), henceforth RR, reach two findings about US COVID-19 vaccination mandates. First, they find that state mandates have no effect on COVID-19 vaccine take-up. Second, they find that compared to states that banned COVID-19 vaccination requirements, states that imposed COVID-19 vaccination mandates exhibit lower adult and child uptake of flu vaccines and lower uptake of COVID-19 boosters. RR interpret these differences causally.

**Fig. 1.**
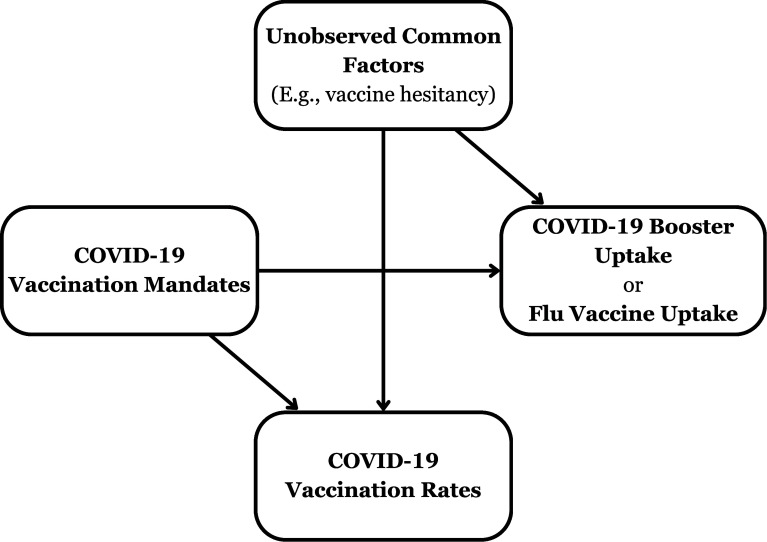
Directed acyclic graph showing the relationships between COVID-19 vaccination mandates, COVID-19 vaccination rates, COVID-19 booster/flu vaccine uptake, and unobserved factors such as vaccine hesitancy.

I focus here on RR’s second set of findings concerning COVID-19 booster and flu vaccine take-up, as these results arise from models exhibiting a key error: They include a bad control (2–4). Specifically, these models control for state COVID-19 vaccination rates. Fig. 1 demonstrates why this is a bad control by showing causal pathways using a directed acyclic graph. A common factor (e.g., vaccine hesitancy) impacts all vaccination rates. Mandates may impact the uptake of COVID-19 boosters or flu vaccines. If mandates also impact COVID-19 vaccination rates, then in models that control for COVID-19 vaccination rates, statistical associations between mandates and COVID-19 booster/flu vaccine uptake will reflect not just causal effects but also *collider bias* (2–4). Though RR find that mandates have insignificant effects on COVID-19 vaccine uptake, they note that they lack enough data to detect small effects. Credible causal analyses with more data show that mandates had strong positive effects on COVID-19 vaccination rates in multiple countries, including the United States (5, 6). In any event, RR’s statistically insignificant mandate effect estimates do not prove that mandates have *zero* effect on COVID-19 vaccination rates (7); this assumption is untestable.

The bad control problem is well established but often ignored (8). Collider bias has impacted scientific conclusions in public health before, including those concerning COVID-19 risk factors (3, 4). For instance, collider bias yielded the “birth weight paradox,” which incorrectly asserts that maternal smoking can be shown to reduce infant mortality after controlling for birth weight (2, 9). I use RR’s data and code (10) to show in Table 1 that collider bias may induce similarly erroneous conclusions in RR’s paper. When the bad control is removed from RR's baseline models (without interaction terms), mandates are no longer negatively associated with COVID-19 booster or flu vaccine take-up. In fact, these associations are significantly positive for both types of vaccine. The replication package for this analysis is publicly available at osf.io/mdfb4/.

**Table 1.** RR’s COVID-19 booster/flu vaccine uptake models with and without the bad control

	COVID-19 booster uptake	Adult flu vaccine uptake	Child flu vaccine uptake
Panel A: Bad control included			
Mandate state	−0.072	−0.119	−0.179
	(0.027)	(0.021)	(0.028)
COVID-19 vaccination rate	3.686 (0.056)	1.518 (0.07)	2.194 (0.029)
N	1,025	205	1,025
Conditional/marginal *R*^2^	0.907/0.55	0.941/0.643	0.975/0.65
Panel B: Bad control removed			
Mandate state	0.045	0.058	0.079
	(0.021)	(0.016)	(0.022)
N	1,025	205	1,025
Conditional/marginal *R*^2^	0.298/0.034	0.512/0.155	0.597/0.151

*Note*: SE are reported in parentheses.

Though average COVID-19 booster and flu vaccine uptake is higher in states that imposed mandates, this does not necessarily mean that mandates *caused* higher uptake. Both RR’s estimates and mine simply reflect the differences in average (conditional) take-up between mandate states and states that banned vaccine requirements. These differences reflect many factors beyond mandates and should not be interpreted causally, even after controlling for COVID-19 vaccination rates. The key conclusion of this replication is that RR’s findings on COVID-19 booster and flu vaccine uptake are not robust, as removing just one control variable can completely reverse these findings. This specific robustness issue is limited to RR’s findings on COVID-19 booster and flu vaccine uptake and does not concern their findings on COVID-19 vaccine uptake.
